# Supplementary material for: Targeted virome deep sequencing reveals frequent herpesvirus detection in intestinal biopsies of inflammatory bowel disease patients
Source: PLoS One. 2025 Dec 11;20(12):e0337322. doi: 10.1371/journal.pone.0337322 (PMC12697960; doi:10.1371/journal.pone.0337322)
Supplement: S1 File — This file contains the following supplementary tables referenced in the manuscript: S1 Table. Virome characteristics (CZID metrics) of 56 intestinal biopsy samples from patients with inflammatory bowel disease (IBD). S2 Table. CZID metrics per sample after quality thresholds. S3 Table. Viral detection by disease type and Fisher–Freeman–Halton permutation p-values. (PDF) [file pone.0337322.s001.pdf]

Supplementary Table S1. Virome characteristics (CZID metrics) of 56 intestinal biopsy samples from patients with inflammatory bowel disease (IBD).

Sample ID	Virus	Score	Z-Score	rPM	Reads	Contigs	Contig Reads	% Identity	Alignment Length (L)	E-value
LMETA1	EBV	37,290,675	1E-0	2,070.0	91206	4	0	99	166.2	1E-78
LMETA2	----	----	----	----	----	----	----	----	----	----
LMETA3	----	----	----	----	----	----	----	----	----	----
LMETA4	CMV	23,122,026	99.0	2,359.1	155,737	0	0	99.3	149.8	1E-98
LMETA4	EBV	52,983,172	1E-0	2,898.8	191,364	1	23,914	99.2	155.2	1E-90
LMETA5	----	----	----	----	----	----	----	----	----	----
LMETA6	PV	9,712,577	1E-0	485.6	16,6	0	0	99.3	120.3	1E-68
LMETA7	CMV	87,073,126	99.0	8,883.4	86,542	7	19,354	99.5	165.8	1E-96
LMETA7	HHV-6	3,885,262	1E-0	162.7	1,585	1	1,098	99.4	228.8	1E-122
LMETA8	EBV	315,776,608	1E-0	17,648	251,233	4	72,893	99.3	191.4	1E-89
LMETA9	CMV	9,057,558	77.1	1,517.3	10,881	0	0	99.2	1E-5.9	1E-59
LMETA10	EBV	569,530,258	1E-0	31,676.4	716,079	19	270,46	99.4	209	1E-122
LMETA10	CMV	349,416,046	99.0	35,581.1	804,35	15	225,447	99.4	184.6	1E-107
LMETA11	----	----	----	----	----	----	----	----	----	----
LMETA12	HHV-7	2,895,491	1E-0	144.8	3,618	0	0	99.4	142.2	1E-83

LMETA12	EBV	5,991,056	1E-0	299.6	7,486	1	7,334	99.9	263.3	1E-137
LMETA13	HADV	47,039	1E-0	2.4	86	0	0	98.5	98.0	1E-52
LMETA14	HCV	8,297,855	1E-0	414.9	15,846	0	0	95.6	127.3	1E-67
LMETA15	----	----	----	----	----	----	----	----	----	----
LMETA16	EBV	40,246,657	1E-0	2,384.3	58,854	3	33,188	99.6	196.9	1E-106
LMETA16	HHV-7	1,1E-1,522	1E-0	55.1	1,359	0	0	99.5	151.1	1E-87
LMETA17	EBV	121,903,239	1E-0	6,63	426,88	10	182,887	99.3	263.2	1E-94
LMETA17	HHV-7	4,409,639	1E-0	220.5	14,198	0	0	99.2	150.5	1E-88
LMETA18	EBV	3,813,615	1E-0	222.3	6,782	1	1,9	99.0	155.4	1E-86
LMETA19	NV	984,457	1E-0	49.2	2,86	0	0	99.4	123.0	1E-70
LMETA20	EBV	126,215,906	1E-0	6,441.6	80,232	0	0	99.3	150.3	1E-88
LMETA20	CMV	40,391,845	99.0	4,121.1	51,33	0	0	99.1	148.8	1E-87
LMETA21	EBV	283,877,064	1E-0	14,948.3	1,556,643	113	1,004,727	99.7	708.1	1E-189
LMETA21	HHV-7	776,1E-9	1E-0	49.4	5,14	1	2,198	99.6	214.3	1E-166
LMETA22	HHV-7	2,817,960	1E-0	140.9	5,946	0	0	99.3	147.3	1E-87
LMETA22	EBV	925,576	1E-0	61.7	2,604	0	0	99.5	150.4	1E-89
LMETA23	EBV	9,275,353	1E-0	618.6	50,198	1	4	99.2	149.6	1E-88
LMETA24	CMV	240,932,214	99.0	24,562.1	411,722	3	155,216	99.6	194.4	1E-127

LMETA25	HHV-6	964,825	1E-0	47.9	858	1	84	99.5	161.2	1E-93
LMETA25	EBV	20,112	1E-0	1	18	0	0	98.2	1E-8.0	1E-58
LMETA26	HHV-6	133,864	1E-0	6.8	146	0	0	99.8	125.0	1E-72
LMETA27	HHV-7	16,345,151	1E-0	859.5	7,073	4	1,605	99.3	178.7	1E-108
LMETA27	HHV-6	465,442	1E-0	35.1	289	1	216	99.6	186.0	1E-101
LMETA27	EBV	2,445,089	1E-0	122.3	1,006	2	974	99.7	273,5	1E-192
LMETA28	EBV	15,446,392	1E-0	1,008.1	5,172	1	1,29	99.4	170.3	1E-80
LMETA28	HHV-6	3,436,213	1E-0	215.6	1,1E-6	2	360	99.0	167.2	1E-92
LMETA29	HHV-6	10,115,586	1E-0	633.3	7,179	4	1,082	99.5	161.6	1E-93
LMETA29	HADV	189,678	1E-0	18.9	214	0	0	99.3	149.0	1E-88
LMETA30	CMV	1,454,225,004	99.0	140,564.0	7,888,763	1E-3	7,847,996	99.8	4,623.5	1E-302
LMETA30	EBV	119,080,791	1E-0	6,229.4	349,61	49	164,436	99.5	253.1	1E-141
LMETA31	----	----	----	----	----	----	----	----	----	----
LMETA32	----	----	----	----	----	----	----	----	----	----
LMETA33	HHV-7	194,826	1E-0	11.3	632	0	0	99.3	145.3	1E-85
LMETA34	HHV-7	407,006	1E-0	22.6	888	0	0	99.3	147.8	1E-87
LMETA35	HHV-6	165,332	1E-0	16.5	952	1	952	1E-0	271.0	1E-139
LMETA36	HHV-7	372,757	1E-0	18.6	492	0	0	99.1	115.0	1E-64

LMETA37	EBV	411,530,340	1E-0	21,467.7	237,08	98	172,205	99.6	651.0	1E-188
LMETA37	HHV-7	565,035	1E-0	28.3	312	0	0	99.7	146.1	1E-187
LMETA38	HHV-7	30,925,300	1E-0	1,672.8	10,163	6	1,755	99.6	180.6	1E-105
LMETA39	HHV-7	34,962,937	1E-0	1,881.2	9,028	4	1,886	99.4	168.8	1E-96
LMETA40	CMV	954,065,202	99.0	74,700.4	1,277,784	174	764,447	99.6	316.7	1E-172
LMETA41	HHV-7	97,886	1E-0	6.5	66	0	0	99.5	151.0	1E-89
LMETA42	----	----	----	----	----	----	----	----	----	----
LMETA43	EBV	2,044,352	1E-0	110.7	11,994	2	2,72	99.4	163.0	1E-92
LMETA43	CMV	993,831	99.0	1E-1.4	10,988	3	2,374	99.5	169.9	1E-96
LMETA43	HHV-7	8,859	1E-0	0.9	96	0	0	98.5	96.5	1E-52
LMETA44	HHV-7	1,012,398	1E-0	50.6	1256	1	1256	99.1	235.0	1E-115
LMETA45	EBV	8,939,783	1E-0	488.4	21,336	4	9,76	99.5	191.6	1E-104
LMETA45	NV	6,254,209	1E-0	312.7	13,662	1	1,524	99.3	160	1E-91
LMETA45	HHV-7	1,991,807	1E-0	144.8	5,016	0	0	99.4	149.5	1E-88
LMETA46	HHV-7	774,585	1E-0	38.7	642	1	194	99.2	197.8	1E-104
LMETA47	EBV	4,1E-9,918	1E-0	205.5	6,19	0	0	99.4	119.9	1E-68
LMETA47	HHV-7	135,149	1E-0	6.8	204	0	0	99.1	151.0	1E-89
LMETA48	CMV	150,347,723	99.0	15,294.9	1,367,421	219	1,033,766	99.6	430.1	1E-211

LMETA48	EBV	16,087,588	1E-0	827.1	73,945	4	19,008	99.3	225.9	1E-131
LMETA48	HHV-7	7,148,026	1E-0	383.7	34,303	4	3,567	99.3	158.0	1E-91
LMETA49	HHV-7	9,198,340	1E-0	478.9	19,112	7	3,44	99.4	165.7	1E-93
LMETA49	HHV-6	2,401,070	1E-0	122.3	4,882	8	2,141	99.6	187.9	1E-103
LMETA50	EBV	3,813,481	1E-0	208.1	10,338	2	1,378	99.3	148.8	1E-85
LMETA50	HHV-7	716,764	1E-0	37.4	1,858	1	126	99.2	149.2	1E-84
LMETA51	HHV-7	1,291,720	1E-0	64.6	1,786	1	1,004	99.7	210.7	1E-115
LMETA52	EBV	2,332,803	1E-0	116.6	4,666	1	4,658	1E-0	275.5	1E-142
LMETA52	HHV-7	1,889,840	1E-0	94.5	3,78	0	0	99.4	148.7	1E-88
LMETA53	HPV	120,046,654	1E-0	50,484.0	2,006,076	2	2,004,429	99.9	3,892.2	1E-307
LMETA53	EBV	14,121,902	1E-0	939.9	37,348	1	14,716	98.5	174.6	1E-96
LMETA54	HHV-7	42,131,204	1E-0	2,170.8	97,149	13	30,892	99.5	183.7	1E-105
LMETA55	HHV-7	315,517	1E-0	15.8	633	0	0	99.2	91.0	1E-49
LMETA56	HHV-7	29,683	1E-0	1.5	78	0	0	99.5	151.0	1E-89

Footnote: EBV, Epstein–Barr virus; CMV, cytomegalovirus; HHV-6/7, human herpesvirus 6/7. Score: Experimental ranking score used to prioritize microbes based on their abundance (rPM) and comparison to controls (Z-score). Z-score: Statistical measure of enrichment relative to background. rPM: Reads aligning per million sequenced reads. Contigs: Number of assembled contigs aligning to the taxon. % Identity: Mean nucleotide identity to reference genomes. L: Mean alignment length. E-value: Mean expectation value of alignments to NCBI NR/NT databases.

Supplementary Table S2. CZID metrics per sample after quality thresholds.

Sample ID	Virus	Score	Z-Score	rPM	Reads	Contigs	Contig Reads	% Identity	Alignment Length (L)	E-value
LMETA1	EBV	37,290,675	1E-0	2,070.0	91206	4	0	99	166.2	1E-78
LMETA4	EBV	52,983,172	1E-0	2,898.8	191,364	1	23,914	99.2	155.2	1E-90
LMETA7	CMV	87,073,126	99.0	8,883.4	86,542	7	19,354	99.5	165.8	1E-96
LMETA7	HHV-6	3,885,262	1E-0	162.7	1,585	1	1,098	99.4	228.8	1E-122
LMETA8	EBV	315,776,608	1E-0	17,648	251,233	4	72,893	99.3	191.4	1E-89
LMETA10	EBV	569,530,258	1E-0	31,676.4	716,079	19	270,46	99.4	209	1E-122
LMETA10	CMV	349,416,046	99.0	35,581.1	804,35	15	225,447	99.4	184.6	1E-107
LMETA12	EBV	5,991,056	1E-0	299.6	7,486	1	7,334	99.9	263.3	1E-137
LMETA16	EBV	40,246,657	1E-0	2,384.3	58,854	3	33,188	99.6	196.9	1E-106
LMETA18	EBV	3,813,615	1E-0	222.3	6,782	1	1,9	99.0	155.4	1E-86
LMETA21	EBV	283,877,064	1E-0	14,948.3	1,556,643	113	1,004,727	99.7	708.1	1E-189
LMETA21	HHV-7	776,1E-9	1E-0	49.4	5,14	1	2,198	99.6	214.3	1E-166
LMETA23	EBV	9,275,353	1E-0	618.6	50,198	1	4	99.2	149.6	1E-88
LMETA24	CMV	240,932,214	99.0	24,562.1	411,722	3	155,216	99.6	194.4	1E-127
LMETA25	HHV-6	964,825	1E-0	47.9	858	1	84	99.5	161.2	1E-93
LMETA27	HHV-7	16,345,151	1E-0	859.5	7,073	4	1,605	99.3	178.7	1E-108

LMETA27	HHV-6	465,442	1E-0	35.1	289	1	216	99.6	186.0	1E-101
LMETA27	EBV	2,445,089	1E-0	122.3	1,006	2	974	99.7	273,5	1E-192
LMETA28	EBV	15,446,392	1E-0	1,008.1	5,172	1	1,29	99.4	170.3	1E-80
LMETA28	HHV-6	3,436,213	1E-0	215.6	1,1E-6	2	360	99.0	167.2	1E-92
LMETA29	HHV-6	10,115,586	1E-0	633.3	7,179	4	1,082	99.5	161.6	1E-93
LMETA30	CMV	1,454,225,004	99.0	140,564.0	7,888,763	1E-3	7,847,996	99.8	4,623.5	1E-302
LMETA30	EBV	119,080,791	1E-0	6,229.4	349,61	49	164,436	99.5	253.1	1E-141
LMETA35	HHV-6	165,332	1E-0	16.5	952	1	952	1E-0	271.0	1E-139
LMETA37	EBV	411,530,340	1E-0	21,467.7	237,08	98	172,205	99.6	651.0	1E-188
LMETA38	HHV-7	30,925,300	1E-0	1,672.8	10,163	6	1,755	99.6	180.6	1E-105
LMETA39	HHV-7	34,962,937	1E-0	1,881.2	9,028	4	1,886	99.4	168.8	1E-96
LMETA40	CMV	954,065,202	99.0	74,700.4	1,277,784	174	764,447	99.6	316.7	1E-172
LMETA43	EBV	2,044,352	1E-0	110.7	11,994	2	2,72	99.4	163.0	1E-92
LMETA43	CMV	993,831	99.0	1E-1.4	10,988	3	2,374	99.5	169.9	1E-96
LMETA44	HHV-7	1,012,398	1E-0	50.6	1256	1	1256	99.1	235.0	1E-115
LMETA45	EBV	8,939,783	1E-0	488.4	21,336	4	9,76	99.5	191.6	1E-104
LMETA45	NV	6,254,209	1E-0	312.7	13,662	1	1,524	99.3	160	1E-91
LMETA46	HHV-7	774,585	1E-0	38.7	642	1	194	99.2	197.8	1E-104

LMETA48	CMV	150,347,723	99.0	15,294.9	1,367,421	219	1,033,766	99.6	430.1	1E-211
LMETA48	EBV	16,087,588	1E-0	827.1	73,945	4	19,008	99.3	225.9	1E-131
LMETA48	HHV-7	7,148,026	1E-0	383.7	34,303	4	3,567	99.3	158.0	1E-91
LMETA49	HHV-7	9,198,340	1E-0	478.9	19,112	7	3,44	99.4	165.7	1E-93
LMETA49	HHV-6	2,401,070	1E-0	122.3	4,882	8	2,141	99.6	187.9	1E-103
LMETA50	EBV	3,813,481	1E-0	208.1	10,338	2	1,378	99.3	148.8	1E-85
LMETA50	HHV-7	716,764	1E-0	37.4	1,858	1	126	99.2	149.2	1E-84
LMETA51	HHV-7	1,291,720	1E-0	64.6	1,786	1	1,004	99.7	210.7	1E-115
LMETA52	EBV	2,332,803	1E-0	116.6	4,666	1	4,658	1E-0	275.5	1E-142
LMETA53	HPV	120,046,654	1E-0	50,484.0	2,006,076	2	2,004,429	99.9	3,892.2	1E-307
LMETA53	EBV	14,121,902	1E-0	939.9	37,348	1	14,716	98.5	174.6	1E-96
LMETA54	HHV-7	42,131,204	1E-0	2,170.8	97,149	13	30,892	99.5	183.7	1E-105

Note: Same parameter definitions as in Supplementary Table S1. Only taxa fulfilling all quality thresholds (Z-score > 75, % identity > 90, rPM > 10, and ≥ 1 contig) are shown.

Supplementary Table S3. Viral detection by disease type and Fisher–Freeman–Halton permutation p-values

Virus	Ulcerative colitis (n = 37)	IBD-U (n = 9)	Ulcerative proctitis (n = 7)	Crohn's disease (n = 3)	Total n (%)	*p* (Fisher perm.)	*q* (FDR)
**EBV**	12	3	4	0	19 (33.9)	*0.234*	*0.402*
**HHV-7**	5	3	3	1	12 (21.4)	*0.286*	*0.446*
**CMV**	4	3	0	0	7 (12.5)	*0.192*	*0.371*
**HHV-6**	6	0	1	0	7 (12.5)	*0.258*	*0.408*
**HPV**	1	0	0	0	1 (1.8)	*0.874*	*0.912*
**NV**	0	0	1	0	1 (1.8)	*0.872*	*0.912*

Footnote: p values were obtained using Fisher–Freeman–Halton permutation tests (20 000 Monte Carlo iterations); *q* values were adjusted for multiple testing using the Benjamini–Hochberg procedure.
