# Supplementary material for: Targeted virome deep sequencing reveals frequent herpesvirus detection in intestinal biopsies of inflammatory bowel disease patients
Source: PLoS One. 2025 Dec 11;20(12):e0337322. doi: 10.1371/journal.pone.0337322 (PMC12697960; doi:10.1371/journal.pone.0337322)
Supplement: Attachment — Submitted filename: Response_to_Reviewer_and_Editor.docx [file pone.0337322.s002.docx]

# Response to Reviewer and Editor Comments

Response to Reviewer and Editor Comments

We would like to sincerely thank the Academic Editor and Reviewer for their thoughtful and constructive feedback on our manuscript “Targeted Virome Deep Sequencing Reveals Frequent Herpesvirus Detection in Intestinal Biopsies of IBD Patients”. We are grateful for the opportunity to revise our work. Below, we provide a detailed, point-by-point response. Reviewer comments are presented in italics, followed by our responses.

We have thoroughly revised our manuscript. This version incorporates an exhaustive review of all sections, with particular emphasis on improving the figures to enhance clarity and to include additional relevant information.

To streamline the main text and improve readability, we have also moved three large tables to the **Supplementary Information** section, where they are now presented as **Supplementary Tables S1–S3**.

We believe these updates have substantially improved the quality, transparency, and presentation of the manuscript,

Sincerely,
**David Tarragó Asensio** (corresponding author)
on behalf of all co-authors
Instituto de Salud Carlos III, Madrid, Spain

Editorial and Journal Requirements

Comment 1: Please ensure that your manuscript meets PLOS ONE’s style requirements.
Response: We have carefully revised the manuscript to comply with PLOS ONE formatting guidelines for title page, affiliations, references, and file naming.

Comment 2: Update the Data Availability statement.
Response: We have revised the Data Availability section to indicate that all raw sequencing data have been deposited in the European Nucleotide Archive (PRJEB101152), in accordance with PLOS ONE data sharing policies. All other supporting information is available within the main text and the supplementary tables.

Comment 3: Ensure references to Figures 1 and 2 in the text.
Response: We have revised the Results section to explicitly refer to Figures 1 and 2 where the corresponding findings are described.

Comment 4: Refer to Table 2 in the text.
Response: We have removed the previous Table 2 from the main text to streamline the manuscript. Part of its content has been integrated into the revised figures to provide clearer visual summaries, while the remaining detailed information has been reorganized into three supplementary tables (**Supplementary Tables S1–S3**) included in the Supporting Information section.

Reviewer #1 Comments

Clinical characteristics of patients (IBD subtype, treatment history, sample size justification).
Reviewer comment: The manuscript does not specify the number of Crohn’s disease (CD) and ulcerative colitis (UC) cases, or treatment history. No statistical basis for sample size is provided.
Response: We thank the reviewer for this helpful comment. We have now included the explicit number of ulcerative colitis (n = 37), Crohn’s disease (n = 3), ulcerative proctitis (n = 7), and IBD-Unclassified (n = 9) cases in both the *Methods* (section 2.1) and *Results* (section 3.1). The revised text also clarifies that no statistical power calculation was performed, as the cohort represents the complete set of available biopsy samples collected during the study period.

Information regarding treatment history has been described in aggregate form and remains subject to data protection regulations. In addition, the updated figures now display this distribution visually to improve clarity and reader comprehension.
*(see lines ≈ 252–275 and 525–540 in the revised manuscript; IBD subtype distribution also shown in Figures 1A and 1B)*

2. Viral nucleic acid enrichment and bioinformatic pipeline details.
Reviewer comment: The design of pan-viral probes, bioinformatic filtering, and sequencing statistics are insufficiently described.
Response: We appreciate this comment and have substantially expanded the *Methods* section to describe the **Twist Bioscience pan-viral probe design**, which targets 3,154 viral families, and to detail the **bioinformatic filtering and quality control pipeline** used. Specifically, we now explain the steps for **host read removal (using the human GRCh38 reference genome), ERCC sequence filtering, adapter trimming, duplicate read removal, and subsampling** implemented within the **Chan Zuckerberg ID (CZ ID) platform**.

We have also added sequencing statistics in the *Results* section, including the **average number of reads per sample**, **percentage of reads passing quality filters**, and **depth of sequencing** to provide transparency regarding dataset quality.

These methodological details are provided in *Methods sections 2.3 and 2.5*, while sequencing metrics are summarized in the *Results (section 3.2)*.
*(see lines ≈ 326–345, 400–460, and 562–580 in the revised manuscript; sequencing statistics also summarized in Figure 2 and Supplementary Table S1)*

3. Basis for quality control thresholds and contamination prevention.
Reviewer comment: Thresholds are not justified and no contamination controls are mentioned.
Response: We thank the reviewer for this valuable observation. The revised *Methods* section now provides a detailed justification of the analytical thresholds applied during metagenomic filtering and taxonomic classification. Specifically, the **Z-score (>75)**, **percentage identity (>90%)**, **reads per million (rPM >10)**, and **≥1 contig** criteria are explained and referenced as standard parameters validated in published virome studies (*Clooney et al., Cell Host Microbe 2019; Zuo et al., Gut 2019; Kalantar et al., Nat Microbiol 2021*). These thresholds correspond to the default confidence filters recommended by the **Chan Zuckerberg ID (CZ ID)** metagenomic platform.

To address the concern regarding contamination prevention, we have incorporated a description of the **six nuclease-free water negative controls** that were processed in parallel through nucleic acid extraction, enrichment, and sequencing. These controls were included in the **CZ ID background model** to statistically correct for environmental or reagent contaminants. Laboratory workflows were carried out in physically separated pre- and post-PCR areas, with dedicated equipment and reagents to minimize cross-contamination.

All this information has been added to the *Methods* under **Bioinformatic Analysis and Sample Processing**, and summarized in the *Results* for transparency.
*(see lines ≈ 445–465 and 585–600 in the revised manuscript; reference to negative controls also noted in Supplementary Table S1)*

4. Stratified analysis by clinical characteristics and coinfection incidence.
Reviewer comment: Results report only virus frequencies without stratification by clinical factors or coinfection incidence.
Response: We thank the reviewer for this constructive comment. In the revised version, we have performed a **stratified analysis** of viral detection according to **IBD subtype (ulcerative colitis, Crohn’s disease, ulcerative proctitis, and IBD-U)** and **treatment status** (where available). These data are now presented in the *Results* section and visually summarized in the updated **Figure 1**, which includes the distribution of viral families and herpesvirus-positive cases stratified by clinical category.

In addition, we have quantified **coinfection incidence**, defining it as the percentage of patients with ≥2 viruses detected in intestinal biopsies. The results of this analysis are now reported in the *Results (section 3.4)*, and the detailed breakdown—including statistical comparisons—is provided in **Supplementary Table S3**, where **Fisher–Freeman–Halton tests** with **Benjamini–Hochberg correction** were applied.

These changes improve the interpretability of the data by linking virome composition to clinical features.
*(see lines ≈ 640–680 and 710–735 in the revised manuscript; data visualized in Figure 1C–E and detailed in Supplementary Table S3)*

5. Interpretation of herpesvirus role and phage–bacteria interactions.
Reviewer comment: The link between herpesvirus detection and IBD is speculative, and phage–bacteria interactions are only cited, not analyzed.
Response: We appreciate this insightful comment. We agree that the relationship between herpesvirus detection and IBD pathogenesis should be interpreted with caution. Accordingly, we have revised the *Discussion* to explicitly state that our findings demonstrate **associations rather than causation**, and that the potential role of herpesviruses should be regarded as **a working hypothesis requiring further validation**. The corresponding paragraph has been rewritten to adopt a more balanced tone and to acknowledge the limitations of our observational design.

In addition, to address the reviewer’s suggestion, we have added a **preliminary exploratory analysis** describing **co-detection events between Caudoviricetes phages and Enterobacteriaceae** in intestinal biopsies. This information has been incorporated in the *Results (section 3.5)* and summarized in **Supplementary Table S3**, providing initial evidence of potential phage–bacteria interactions within the dataset.

These modifications strengthen the discussion and contextualize the findings within a realistic framework.
*(see lines ≈ 770–820 and 835–855 in the revised manuscript; new co-detection data shown in Figure 2E–F and Supplementary Table S3)*

6. Statement that “herpesviruses are absent in healthy individuals” is too absolute.
Reviewer comment: This claim is overstated and may mislead readers.
Response: We thank the reviewer for this important observation. We have revised the statement in the *Discussion* to avoid overgeneralization and to more accurately reflect current evidence. The sentence now reads:

“Herpesviruses are **rarely detected in healthy intestinal viromes** and are generally considered absent, whereas their frequent detection in IBD biopsies suggests possible pathological relevance.”

This revised phrasing maintains the intended contrast between healthy and IBD cohorts while avoiding categorical statements. The change improves scientific accuracy and aligns the discussion with published data on the prevalence of latent herpesviruses in gut tissues.
*(see lines ≈ 860–880 in the revised manuscript)*

Overall

**Overall Statement**

We have addressed all reviewer and editor comments through substantial revisions to the *Methods*, *Results*, and *Discussion* sections, while also clarifying the study’s limitations. These changes have strengthened the manuscript, improved its clarity and rigor, and ensured full compliance with PLOS ONE’s editorial standards.
